# Huntingtin Is Critical Both Pre- and Postsynaptically for Long-Term Learning-Related Synaptic Plasticity in *Aplysia*

**DOI:** 10.1371/journal.pone.0103004

**Published:** 2014-07-23

**Authors:** Yun-Beom Choi, Beena M. Kadakkuzha, Xin-An Liu, Komolitdin Akhmedov, Eric R. Kandel, Sathyanarayanan V. Puthanveettil

**Affiliations:** 1 Department of Neuroscience, College of Physicians and Surgeons of Columbia University, New York, New York, United States of America; 2 Department of Psychiatry, College of Physicians and Surgeons of Columbia University, New York, New York, United States of America; 3 Howard Hughes Medical Institute, College of Physicians and Surgeons of Columbia University, New York, New York, United States of America; 4 Kavli Institute for Brain Science, College of Physicians and Surgeons of Columbia University, New York, New York, United States of America; 5 Department of Neuroscience, The Scripps Research Institute, Scripps Florida, Jupiter, Florida, United States of America; Texas A&M University - Corpus Christi, United States of America

## Abstract

Patients with Huntington’s disease exhibit memory and cognitive deficits many years before manifesting motor disturbances. Similarly, several studies have shown that deficits in long-term synaptic plasticity, a cellular basis of memory formation and storage, occur well before motor disturbances in the hippocampus of the transgenic mouse models of Huntington’s disease. The autosomal dominant inheritance pattern of Huntington’s disease suggests the importance of the mutant protein, huntingtin, in pathogenesis of Huntington’s disease, but wild type huntingtin also has been shown to be important for neuronal functions such as axonal transport. Yet, the role of wild type huntingtin in long-term synaptic plasticity has not been investigated in detail. We identified a huntingtin homolog in the marine snail *Aplysia*, and find that similar to the expression pattern in mammalian brain, huntingtin is widely expressed in neurons and glial cells. Importantly the expression of mRNAs of huntingtin is upregulated by repeated applications of serotonin, a modulatory transmitter released during learning in *Aplysia*. Furthermore, we find that huntingtin expression levels are critical, not only in presynaptic sensory neurons, but also in the postsynaptic motor neurons for serotonin-induced long-term facilitation at the sensory-to-motor neuron synapse of the *Aplysia* gill-withdrawal reflex. These results suggest a key role for huntingtin in long-term memory storage.

## Introduction

Huntington’s disease (HD) is caused by a mutation that expands the number of trinucleotides CAG repeats in a gene leading to an expansion of polyglutamine stretch in huntingtin, the encoded protein (The Huntington’s Disease Collaborative Research Group, 1993). HD is a neurodegenerative disorder characterized by involuntary movements, emotional disturbance, and cognitive impairment [1]. In HD, early cognitive deficits occur many years prior to overt motor deficits [2], a finding also observed in a transgenic mouse model of HD [3]. At the cellular level, synaptic dysfunction is noted many years before the neuronal cell loss characteristic of neurodegenerative diseases [4], [5]. In various transgenic mouse models of HD, there is a deficit in forms of synaptic plasticity thought to contribute to learning and memory. Specifically, transgenic mice containing mutant huntingtin exhibit reduced long-term potentiation (LTP) as well as an abnormal development of NMDA-dependent long-term depression (LTD) in the hippocampus [6]–[9].

Because of the dominant inheritance pattern of HD, investigation of the pathogenesis of HD has been focused on the mutant huntingtin’s gain-of-function. However, huntingtin is highly conserved from *Drosophila* to humans, suggesting that it likely has a central role in cell biological functions of the nervous system and there may be loss-of-function from the reduced wild type protein that also contributes to HD pathogenesis. Indeed, various experimental approaches have been used to investigate wild type huntingtin function and itss possible involvement in the pathogenesis of HD [10]–[12]. The findings suggesting the role of wild type huntingtin in the pathogenesis of HD include: (1) increased wild type huntingtin expression leads to improved brain cell-survival [13]–[15] and (2) a removal of the wild type huntingtin generates some of the phenotypes observed in the presence of mutant huntingtin such as neuronal cell death [16].

Huntingtin-knockout mice exhibit embryonic death before day 7.5 suggesting that huntingtin is essential for embryonic development [17]–[19]. In post-mitotic neurons, it has a scaffolding function and a possible role as a facilitator of signal transduction [20]. Huntingtin interacts postsynaptically with N-methyl D-aspartate receptors (NMDARs) indirectly by binding to SH3 domain of PSD95, an adaptor protein in the postsynaptic density [21]. Huntingtin is also present presynaptically where it is associated with recycling endosomes, the endoplasmic reticulum, the Golgi complex, and clathrin-coated vesicles and synaptic vesicles [22]–[24]. Increased expression of wild type huntingtin caused an increased transcription of brain-derived neurotrophic factor (BDNF) in mice [25], [26]. *In vitro*, wild type huntingtin stimulates BDNF vesicle trafficking in neuronal cells [27]. Neuronal deletions of *Drosophila* huntingtin using RNAi caused axonal blockage [28], which is characteristic of mutations not only in cytoskeletal motor proteins such as kinesin or dynein that are required for axonal transport, but also proteins that function as binding partners for motor proteins [29], [30]. Huntingtin-associated protein-1 also interacts directly with kinesin light chain [31].

The roles of huntingtin in BDNF production and vesicular transport suggest that wild type huntingtin could be important for learning-related synaptic plasticity. However, despite the results showing dysfunction in LTP and LTD in the brains of transgenic mice expressing mutant huntingtin [6]–[9], the role of wild type huntingtin in long-term learning-related synaptic plasticity has not been studied in detail.

To explore the role of normal huntingtin in long-term learning-related synaptic plasticity, we turned to an elementary neural circuit that underlies a simple form of learned fear in *Aplysia*–sensitization of the gill-withdrawal reflex. Specifically, a critical component of the *Aplysia* gill-withdrawal reflex that contributes importantly to the behavior is a direct monosynaptic connection from the siphon sensory neurons to the gill motor neurons. The sensory-to-motor neuron synapse can be reconstituted in dissociated cell culture and is modulated, as in the intact animal, by serotonin (5-HT), a modulatory transmitter released during the learning of fear [32]. In the sensory-to-motor neuron synapses, one brief application of 5-HT produces short-term facilitation (STF) that lasts minutes, while five spaced applications of 5-HT to these synapses produce long-term facilitation (LTF) that lasts for days and results in growth of new synaptic connections [33], [34]. These identified neurons are larger in size and form precise connections with one another facilitating the study of cell biology of huntingtin in specific cells and cellular compartments at high resolution and allowing selective manipulation of either the presynaptic sensory neuron or postsynaptic motor neuron [35]. Previously, *Aplysia* sensory-to-motor neuron synapse as a model system has been used to show that an overexpression of the mutant human huntingtin N-terminal fragment containing 150 glutamine residues tagged with enhanced green fluorescent protein (Nhtt150Q-EGFP) in sensory neurons inhibits 5-HT induced LTF [36].

In this study, we identified a homolog of huntingtin in *Aplysia.* We find that repeated applications of 5-HT upregulate huntingtin transcripts. Furthermore, knocking down huntingtin mRNAs, in either pre- or postsynaptic neurons abolish 5-HT-induced LTF at the sensory-to-motor neuron synapse, but it did not affect STF. Our findings suggest that huntingtin participates in both pre- and postsynaptic regulation of long-term synaptic plasticity that underlies long-term memory.

## Results

### *Aplysia* homolog of huntingtin (ApHTT)

**Figure 1:**
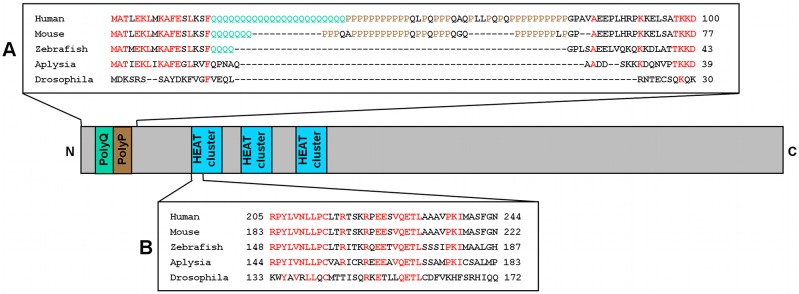
Sequence comparison between an *Aplysia* homolog of huntingtin (ApHTT) and huntingtin from other species. Comparison of the deduced amino acid sequences of ApHTT with huntingtins from other species at (A) the N-terminal end and (B) the first HEAT repeat region. Red letters denote identical amino acid residues. Sequences are from NCBI protein database. Human: NP_002102.4, Mouse: NP_034544.1, Zebrafish: NP_571093.1, *Aplysia*: XP_005093645.1, *Drosophila*: NP_651629.1. The domain structure of human huntingtin is shown as a reference. PolyQ: polyglutamine stretch, PolyP: polyproline region, HEAT cluster: clusters of HEAT repeats.

Screening the *Aplysia* sequence base (www.aplysiagenetools.org) and the NCBI transcript data base yielded a transcript corresponding to a huntingtin homolog in *Aplysia californica* (accession number: XM_005093588.1). The predicted protein, the *Aplysia* homolog of huntingtin (ApHTT) is 2873 amino acids in length, slightly shorter than human huntingtin (3144 amino acids). Comparison of ApHTT with human huntingtin at the amino acid level reveals that ApHTT is 40% identical to human huntingtin (Figure 1). ApHTT does not have the N-terminal polyglutamine stretch, which is expanded in HD, but much shorter in lower vertebrate and absent in *Drosophila* as in *Aplysia* [37], [38]. ApHTT, similar to *Drosophila* and lower vertebrates, also lacks the polyproline region that follows polyglutamine stretch in human or higher vertebrates. However, ApHTT has a high degree of sequence conservation in the first 17 amino acids–12 out of 17 amino acids are identical to human huntingtin – that determine sub-cellular localization and aggregation [39]. In addition, ApHTT has a high degree of sequence conservation in the region of HEAT (Huntingtin, elongation factor 3, regulatory A submit of protein phosphatase 2a and TOR1) repeats, which cluster in three domains in the N-terminal half of human huntingtin, and is thought to be involved in protein-protein interactions [40].

### ApHTT is expressed in presynaptic and postsynaptic neurons in *Aplysia*

**Figure 2:**
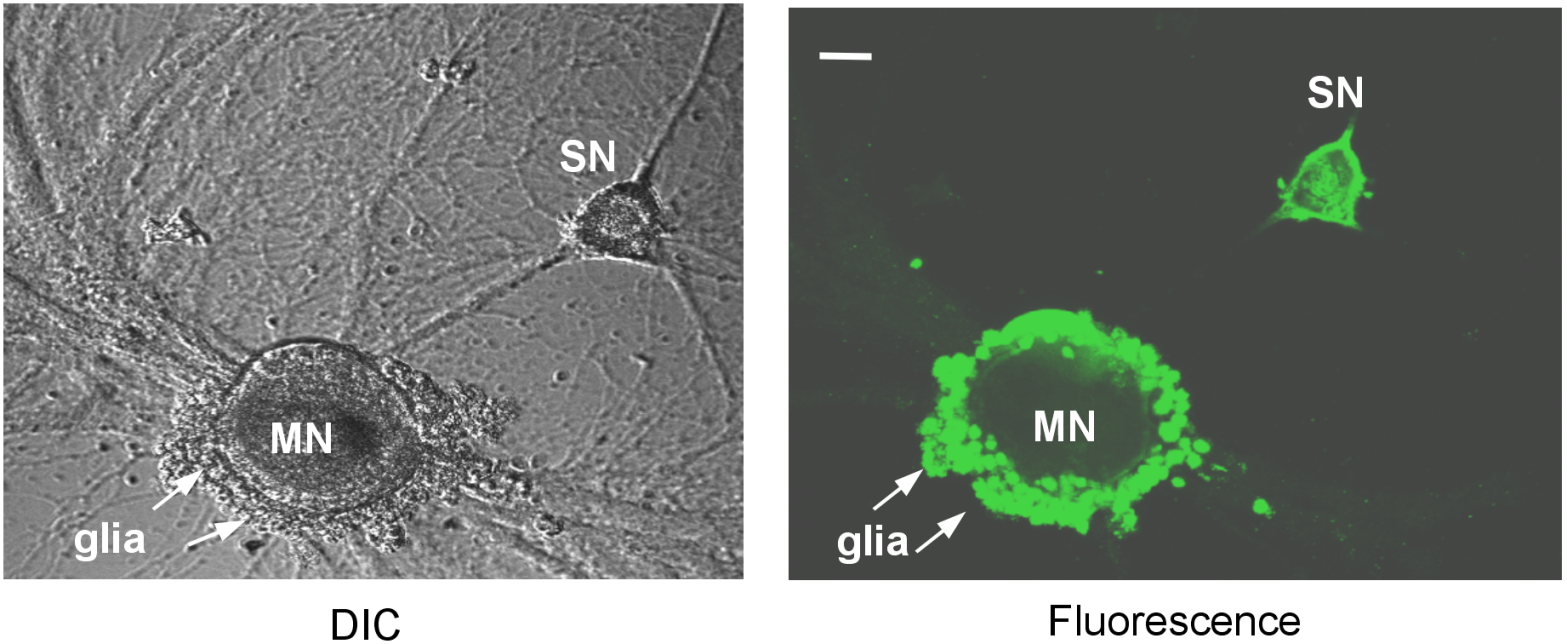
ApHTT mRNAs are expressed in *Aplysia* neurons. mRNA *in situ* analysis shows that ApHTT mRNAs are expression in *Aplysia* sensory neurons, motor neurons, and glial cells. Fluorescently labeled ribo probes were used to examine the distribution of ApHTT mRNAs. Confocal projection image is shown. The scale bar represents 20 µm.

We first examined the distribution of ApHTT mRNAs in sensory-to-motor neuron co-cultures. Based on the ApHTT transcript sequence information, we sub-cloned a 400 base pair fragment and prepared digoxegenin (DIG) labeled antisense ribo-probes. These probes were used in the mRNA *in situ* hybridization experiment to visualize distribution of ApHTT mRNAs. Consistent with the findings on huntingtin distribution in mammalian brain, we find that ApHTT mRNA is ubiquitously expressed in *Aplysia* sensory neurons, motor neurons and in glial cells (Figure 2). ApHTT mRNA is mostly localized in the cell body cytosol of sensory and motor neurons.

### ApHTT mRNAs are induced by repeated applications of 5-HT

**Figure 3:**
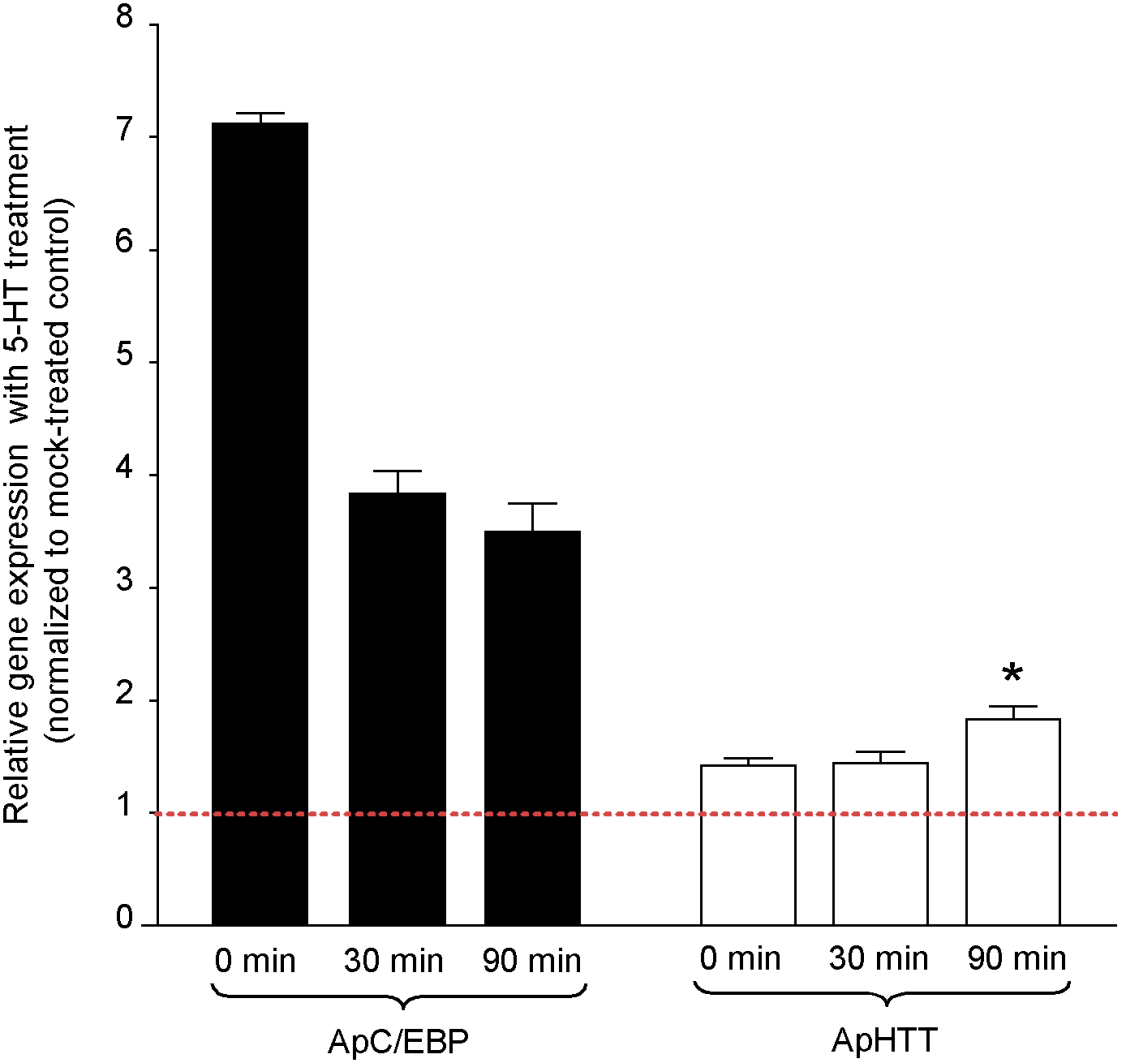
ApHTT mRNAs are induced by 5-HT. RNAs were isolated from pleural sensory neuron clusters at 0, 30 and 90**minutes after the end of five pulses of 5-HT treatment. qRTPCR analysis of RNA is shown in bar graphs. Data was first normalized to 18S rRNA levels. Each bar corresponds to gene expression ratio (5×5-HT treated/mock treated controls). ApC/EBP was used as a positive control. Error bars are SEM.

Transcriptional changes in expression of specific genes are an important component of long-term memory in addition to changes in translation and axonal transport [41]. As a first step to understand the role of ApHTT in memory storage, we used specific primers in qRTPCR reactions to determine whether the transcript levels of ApHTT would change in response to repeated applications of 5-HT (five pulses of 10**µM). We isolated RNAs from pleural sensory neuron clusters at 0, 30, and 90**minutes after the completion of the 5-HT treatment and quantitated changes in ApHTT mRNA levels. We used expression changes in *Aplysia* CCAAT enhancer-binding protein (ApC/EBP) mRNA as a positive control [42]. As expected we found a robust increase in ApC/EBP transcript levels immediately after the 5-HT treatment, which declines gradually over 90**minutes. In contrast, there were no significant changes in ApHTT expression immediately or at 30**minutes after 5-HT treatment. However, there was a significant increase in ApHTT transcript levels at 90**minutes (Figure 3, fold changes: at 0**minute: ApC/EBP 7.10±0.09, p = 0.0002, t = 9.0156, df = 6; ApHTT 1.41±0.07, p = 0.24, t = 1.283, df = 6; at 30**minutes: ApC/EBP 3.80±0.20, p = 0.0011, t = 5.8932, df = 6; ApHTT 1.44±0.09, p = 0.06, t = 1.8266, df = 8; at 90**minutes: ApC/EBP 3.50±0.26, p = 0.0028, t = 3.5775, df = 6; ApHTT 1.81±0.11, p = 0.01, t = 3.5775, df = 6, Student’s t test) suggesting that 5-HT induces a delayed expression of the ApHTT transcripts.

**Figure 4:**
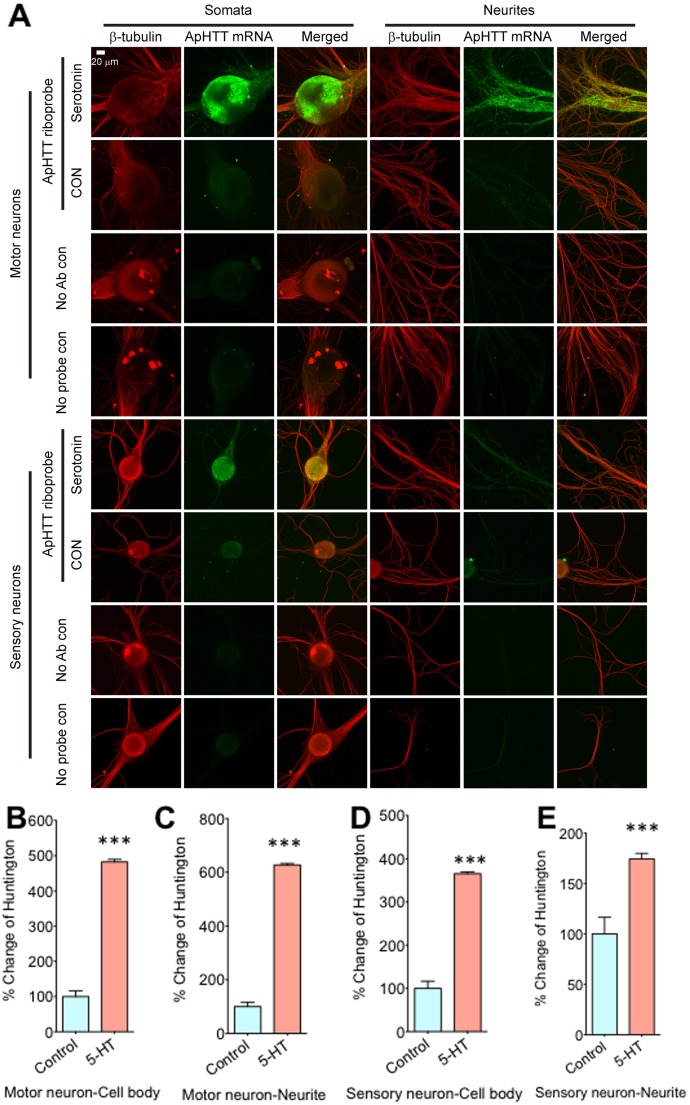
ApHTT mRNAs are induced both in presynaptic sensory neurons and in postsynaptic motor neurons by 5-HT. *Aplysia* sensory-to-motor neuron co-cultures were treated with 5×5-HT (10 µM, 5 minutes) and RNA *in situ* hyrbridization was carried out using riboprobes. A: Confocal projection images showing cell bodies and neurites of sensory neurons and motor neurons. Tubulin protein immunostaining was used visualizing major axon and neurites. No antibody (anti-DIG antibody) and no probe were used as controls for *in situ* hybridization. B, C, D and E: Quantitation of imaging data. % change in fluorescence compared to back ground are shown in bar graphs. Data was normalized to mock treated controls. Error bars are SEM.

We next carried out RNA *in situ* hybridization experiments using ribo probes in sensory-to-motor neuron co-cultures to confirm qRTPCR findings and to examine whether the upregulation of ApHTT occurs only in sensory neurons or both in sensory neurons and in motor neurons. The possibility that 5×5-HT regulated ApHTT in both pre- and post synaptic neurons will further inform us about function of huntingtin in neural circuits. We find that ApHTT mRNA expression is induced both in the cell body and neurites of sensory neurons and motor neurons at 90 minutes after 5-HT treatment (Figure 4, % change when compared to control: Soma, motor neuron: 482.42±7.13%, t = 8.9364, df = 5, p = 0.0004; neurites, motor neuron: 626.95%±5.41% t = 14.0046, df = 6, p = 0.001; soma, sensory neuron: 365.42%±4.00%, t = 12.3577, df = 6; neurites, sensory neuron: 174.30±12.49%, t = 3.8020, df = 6, p = 0.0001 for both soma and neurites, N = 4 for all except for soma of control motor neuron where N = 3).

### Injection of ApHTT anti-sense oligonucleotides into the presynaptic sensory neuron does not affect STF

**Figure 5:**
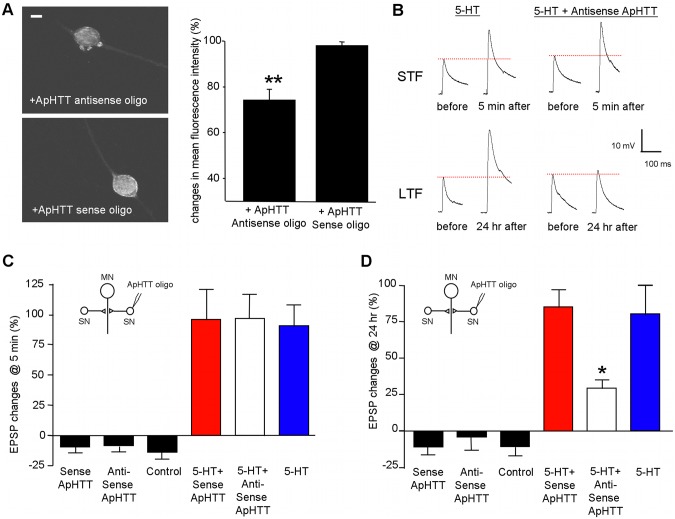
ApHTT in presynaptic sensory neurons is required for the initiation of LTF. A: RNA *in situ* hybridization at 3 hours after oligonucleotides injection. Injection of ApHTT anti-sense oligonucleotides into the presynaptic sensory neuron lead to a decrease in ApHTT mRNA level. Confocal projection images are shown. The scale bar represents 20 µm. The summary bar graph is on the right (N = 8). B: Representative traces of EPSPs that were recorded from motor neurons in response to extracellular stimulation of sensory neurons before and 5 minutes after exposure to 1×5-HT (10 µM, 5 minutes) for STF or before and 24 hours after exposure to 5×5-HT (10 µM, 5 minutes) for LTF. C: Injection of ApHTT anti-sense oligonucleotides in the presynaptic sensory neuron has no effect on STF. D: Injection of ApHTT anti-sense oligonucleotides blocks LTF. Changes in EPSP amplitudes are shown in bar graphs. Error bars are SEM. SN: sensory neuron, MN: Motor neuron.

We next turned to study role of ApHTT in learning-related synaptic plasticity. We used antisense oligonucleotides to knock down ApHTT transcripts in sensory-to-motor neuron cultures in which two sensory neurons make functional synaptic connections to one L7 motor neuron. For all the studies, we injected phosphothio-modified antisense oligonucleotides into one sensory neuron and the other sensory neuron received control oligonucleotides (sense oligonucleotides) or untreated. Microinjection of ApHTT antisense oligonucleotides (50 ng/µl) in presynaptic sensory neurons resulted in a 25±4% (Student’s t test, p<0.01, n = 8) reduction in ApHTT mRNA level compared to the uninjected controls when cultures were fixed at 3 hours after the injections (Figure 5A). Sense oligonucleotides (50 ng/µl) injected into the other sensory neuron in the co-culture as a control did not decrease the level of ApHTT mRNA.

Having established that antisense oligonucleotides are able to knock down ApHTT mRNA levels in sensory neurons, we examined whether the down regulation of ApHTT mRNA by antisense oligonucleotides in the presynaptic sensory neurons affects basal synaptic transmission in the sensory-motor neuron synapse by measuring excitatory postsynaptic potentials (EPSPs) at 24 hours after oligonucleotides injection (50 ng/µl) to the presynaptic sensory neurons. (Figure 5D; % change in EPSP amplitude: no injection −10.0±6.0, n = 7; antisense oligo alone −3.4±9.6, n = 7; sense oligo alone −10.2±5.7, n = 8). One-way ANOVA (F = 0.28, p = 0.76, df = 21) revealed that a 25% reduction in ApHTT mRNA levels does not affect basal synaptic transmission.

We next studied the effect of ApHTT knock down on STF. At 3 hours after injection of the oligonucleotides into presynaptic sensory neurons, we treated cultures with one pulse of 5-HT (10 µM) for five minutes to induce STF. We measured the EPSPs again at 5 minutes after the 5-HT treatment (Figure 5B and C; % change in EPSP amplitude: no injection –13.2±5.9, n = 8; antisense oligo alone –8.1±4.9, n = 13; sense oligo alone −8.7±5.1, n = 11; 5-HT 90.7±17.8, n = 10; 5-HT + antisense 96.6±20.0, n = 12; 5-HT + sense 95.8±25.7, n = 9). One-way ANOVA revealed there were no significant differences among different 5-HT treated groups (F = 0.023, p = 0.98, df = 30). Thus, injection of the antisense oligonucleotides to ApHTT into the presynaptic sensory neuron did not block STF.

### Injection of ApHTT anti-sense oligonucleotides into the presynaptic sensory neuron blocks LTF

We next evaluated the possible presynaptic role of ApHTT in LTF. At 3 hours after initial measurements of EPSPs and injection of the antisense oligonucleotides to ApHTT in the presynaptic sensory neuron, we treated cultures with five repeated pulses of 5-HT (10 µM, 5 minutes) and measured EPSPs again at 24 hours after 5-HT treatment. The injection of the antisense oligonucleotides to ApHTT into presynaptic sensory neurons led to a significant reduction of LTF at 24 hours, but the injection of sense oligonucleotides did not have any significant effect on LTF (Figure 5B and D; % change in EPSP amplitude: 5-HT 80.5±20.8, n = 19; 5-HT + antisense 29.7±6.5, n = 22, 5-HT + sense 85.7±12.8, n = 19, one-way ANOVA: F = 5.10, p = 0.0092, df = 59, followed by Tukey HSD post-hoc test: p<0.05 for 5-HT versus 5-HT + antisense, no significance for 5-HT versus 5-HT + sense). These results, showing that depletion of ApHTT in the presynaptic sensory neuron blocks LTF, support the notion that ApHTT is an important regulatory component of long-term memory storage.

### Injection of ApHTT anti-sense oligonucleotides into the postsynaptic motor neuron does not affect STF

**Figure 6:**
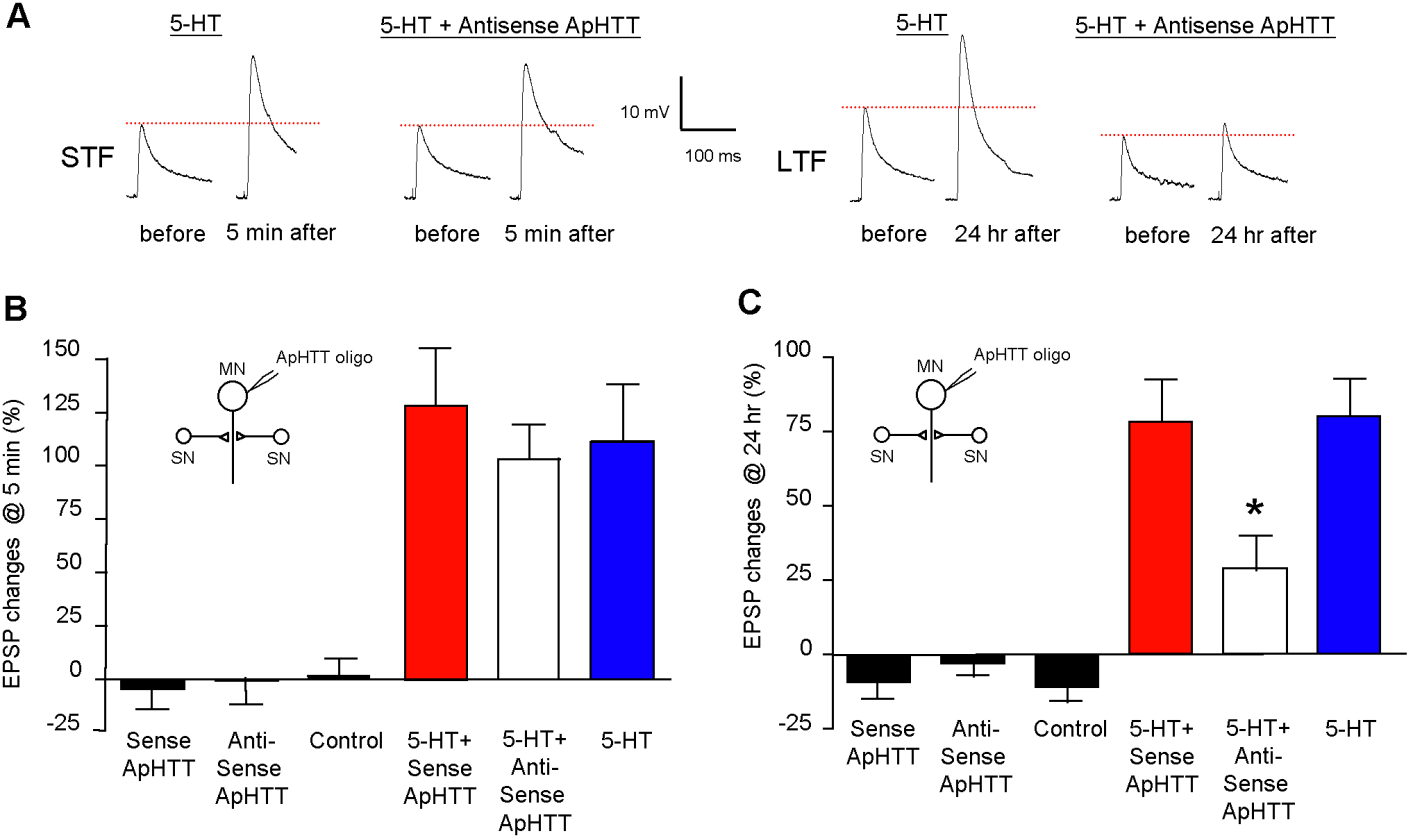
ApHTT in the postsynaptic motor neurons is required for the initiation of LTF. A: Representative traces of EPSPs that were recorded from motor neurons in response to extracellular stimulation of sensory neurons before and 5 minutes after exposure to 1×5-HT (10 µM, 5 minutes) for STF or before and 24 hours after exposure to 5×5-HT (10 µM, 5 minutes) for LTF. B: Injection of ApHTT anti-sense oligonucleotides in the postsynaptic motor neuron has no effect on STF. C: Injection of ApHTT anti-sense oligonucleotides blocks LTF. Changes in EPSP amplitudes are shown in bar graphs. Error bars are SEM. SN: sensory neuron, MN: Motor neuron.

We next examined the role of ApHTT in postsynaptic motor neurons as ApHTT mRNA is present in motor neurons as seen in Figure 2. We first tested whether knockdown of ApHTT mRNAs has any effect on STF. At 3 hours after oligonucleotides injections into the postsynaptic motor neurons, we measured basal EPSPs, then treated cultures with one pulse of 5-HT (10 µM) for five minutes, and again EPSPs were measured at 5 minutes after the 5-HT treatment (Figure 6A and B; % change in EPSP amplitude: no injection 0.8±9.0, n = 6; antisense oligo alone –0.7±11.4, n = 7; sense oligo alone –4.4±10.6, n = 6; 5-HT 112.1±26.2, n = 10; 5-HT + antisense 103.7±16.2, n = 11; 5-HT + sense 129.0±26.6, n = 9). One way ANOVA revealed there were no significant differences among different 5-HT treated groups (F = 0.31, p = 0.74, df = 29). Thus, the injection of antisense oligonucleotides into postsynaptic motor neurons did not affect STF.

### Injection of ApHTT anti-sense oligonucleotides into the postsynaptic motor neuron blocks LTF

Next, we examined whether ApHTT also plays a role in long-term synaptic plasticity in the postsynaptic neurons. At 3 hours after initial measurements of EPSPs and injection of the antisense oligonucleotides to ApHTT (50 ng/µl) in the postsynaptic motor neuron, we treated cultures with five pulses of 5-HT (10 µM) and measured EPSPs again at 24 hours after 5-HT treatment. Similar to our results in presynaptic sensory neurons, we find that basal synaptic transmission was not affected by the antisense oligonucleotides injections that knock down ApHTT mRNAs (Figure 6A and C; % change in EPSP amplitude: no injection –10.7±4.9, n = 8; antisense oligo alone –2.8±4.0, n = 9; sense oligo alone −8.9±5.8, n = 8, one way ANOVA: F = 0.73, p = 0.49, df = 24). In contrast, injection of the antisense oligonucleotides to ApHTT leads to a significant reduction of LTF at 24 hours, but the injection of sense oligonucleotides did not have any significant effect on LTF (Figure 6A and C; % change in EPSP amplitude: 5-HT 80.1±13.1, n = 15; 5-HT + sense 77.9±14.8, n = 17; 5-HT + antisense 28.7±11.7, n = 13, one way ANOVA: F = 4.22, p = 0.022, df = 44, followed by Tukey HSD post-hoc test, : p<0.05 for 5-HT versus 5-HT + antisense, no significance for 5-HT versus 5-HT + sense). Taken together, these results show that the depletion of ApHTT in the postsynaptic motor neurons blocks the establishment of LTF whereas STF was unaffected by antisense oligonucleotides injections.

## Discussion

Animal models such as *Drosophila* [43] zebrafish [44] and rodents [45] have been useful in obtaining important insights into HD. For example, mouse models of HD that express full-length human, or full-length mouse mutant huntingtin have been studied [46]. However, very few studies have examined regional- or temporal-specific knockdown of huntingtin or overexpression of mutant huntingtin. A study found that a knockdown of huntingtin expression using shRNAs in neuroepithelial cells of neocortex led to disturbed cell migration, reduced proliferation, and increased cell death in ways that are relatively specific to early neural development [47]. Interestingly, this study also found that huntingtin knockdown results in cell death but not perturbed migration in the cerebellum, suggesting region-specific functions of huntingtin. In another study, reducing huntingtin mRNA levels transiently in a mouse model of HD using specific antisense oligonucleotides has reversed disease phenotypes such as cell death [48]. Even in these studies where a temporal control of knockdown is achieved, different neuronal populations including interneurons as well as non-neuronal cells such as glia are manipulated at the same time. Moreover, none of these earlier studies examined the selective role of huntingtin in pre- and postsynaptic compartments. As a result, we chose to study the sensory-to-motor neuron synapse of the *Aplysia* gill-withdrawal reflex reconstituted in culture in order to examine the function of normal huntingtin in memory storage. In *Aplysia*, selective manipulation of the presynaptic sensory neurons and postsynaptic motor neurons is readily manageable and addressing this issue seemed important because long-term memory storage is associated with specific and coordinated pre- and postsynaptic changes [35].

As the first step in investigating the role of huntingtin at the sensory-to-motor neuron synapse of *Aplysia* gill-withdrawal reflex, we identified the *Aplysia* homolog of huntingtin from the database. In wild type human huntingtin, the length of the N-terminal polyglutamine stretch is on average 18 amino acids and when the expansion of the polyglutamine stretch reaches to be greater than 37, it causes HD (The Huntington’s Disease Collaborative Research Group, 1993). However, in mice huntingtin has seven glutamines, zebrafish huntingtin has only four glutamines, and *Drosophila* huntingtin has no glutamine stretch. Similar to *Drosophila* huntingtin, ApHTT does not have a polyglutamine stretch nor adjacent polyproline region. Thus, the polyglutamine stretch may not be required for the normal biological function of huntingtin [37]. Importantly ApHTT has high conservation in the region corresponding to the region of HEAT repeats clusters in the N-terminal of human huntingtin. Since HEAT repeats are important for normal huntingtin functions including cellular transport by mediating protein-protein interactions [40], ApHTT may have similar protein interacting partners as human huntingtin. In addition, consistent with data from huntingtin expression in other animals such as mouse and zebrafish, ApHTT mRNAs are ubiquitously expressed in presynaptic sensory neurons, postsynaptic motor neurons and glial cells.

During memory storage in *Aplysia,* transcription of several genes are upregulated in response to 5-HT exposure (Puthanveettil and Kandel, 2011). Most of the known genes that are transcriptionally upregulated by 5-HT are immediate early genes and the upregulation occurs within one hour of repeated 5-HT exposure. These genes include ApC/EBP [42], *Aplysia* kinesin heavy chain 1 (ApKHC1) and *Aplysia* kinesin light chain 2 (ApKLC2) [41]. Very few genes that are upregulated late in response to 5-HT treatment are known. For example, *Aplysia* eukaryotic translation elongation factor 1 alpha Ap (ApEF1 alpha) is upregulated by 4–6 hrs after 5-HT treatment [49]. Our qRTPCR data showed that there were no significant changes in ApHTT transcript levels immediately or at 30 minutes after 5-HT treatment. However, at 90 minutes after 5-HT treatment, we find significant upregulation of ApHTT. These results suggests that ApHTT mRNA levels are transcriptionally regulated as a late gene when compared to ApC/EBP and ApKHC1 during long-term memory storage. Furthermore our *in situ* hybridization analysis suggested that the transcriptional upregulation occurs both in presynaptic sensory neurons as well as postsynaptic motor neurons. This upregulation in both components of the the circuitry suggested a potential role in mediating long-term synaptic plasticity and memory storage.

To understand the role of ApHTT in long-term memory storage, we knocked down ApHTT mRNAs using specific phosphothio-modified antisense oligonucleotides. Injection of antisense oligonucleotides in either pre- or postsynaptic neurons inhibited LTF induced by 5 pulses of 5-HT without affecting basal synaptic transmission or STF. Interestingly, this phenotype is similar to what we observed previously that ApKHC1 knockdown in either pre- or postsynaptic neurons did not affect STF, but blocked the initiation of LTF [41]. Based on these results, we previously suggested that kinesin transport in the postsynatic motor neuron is important for the initiation of LTF and associated synaptic growth in both pre- and postsynaptic compartments and that these may be regulated by coordinated transynaptic signaling between the two compartments. In support of this idea, we have shown previously that transynaptic interaction of postsynaptic neuroligin with presynaptic neurexin is important for initiation of LTF and associated growth of new synaptic connection in the sensory-to-motor neuron synapse of the *Aplysia* gill-withdrawal reflex [50]. Both neurexin and neuroligins are protein cargos transported by ApKHC1 [41]. Since huntingtin may play a role in cellular trasnport [27] [28], hungtingtin along with kinesin motor may mediate one of critical pre and postsynpatic steps for the initiation of LTF.

Another possible mechanism that can explain the observed electrophysiological phenotype is the proposed role of huntingtin in BDNF production. Overexpression of wild type huntingtin increases BDNF protein levels *in vitro* and *in vivo* by regulating the BDNF gene transcription [25], [26]. Moreover, huntingtin knockdown in zebrafish by antisense oligonucleotides leads to a reduction of BDNF expression [51]. Neurotrophins in general and BDNF in particular, have important roles in neuronal survival and synaptic plasticity [52]. Indeed, BDNF has been shown to reverse LTP deficit in knock-in mouse model of HD [6]. We recently showed a neurotrophin and its receptor Trk (ApNT and ApTrk) are present in *Aplysia* and they are important for 5-HT induced LTF [53]. Huntingtin could also be involved in transcriptional regulation of genes other than BDNF important for long-term synaptic plasticity since it also interacts with transcription factors such as cAMP response-element binding protein (CREB)-binding protein (CBP) [54]. Knockdown of huntingtin may disrupt the transcription apparatus required for long-term synaptic plasticity.

Previously, an overexpression of the mutant human huntingtin N-terminal fragment containing 150 glutamine residues tagged with enhanced green fluorescent protein (Nhtt150Q-EGFP) in sensory neurons of the *Aplysia* sensory-to-neuron synapse impaired LTF indueced by repeated pulses of 5-HT without affecting basal synaptic transimssion or STF [36]. The same electrophysiological phenytypes observerd in our study using the knockdown of the ApHTT further support the idea that both the gain-of-function from the mutant huntington and the loss-of-function from the reduction of wild type huntingting may play a role in congnitive deficit in patients with HD.

One major limitation of our study is that we were not able to characterize endogenous ApHTT because of a lack of antibodies against ApHTT. Atlhough the half-life of ApHTT protein is not known, given the robust electrophysioolgical phyenotyes we observed with antisense oligonucleotide injections, we have made the assumption that a 25% decrease in mRNA at 3 hours post injection would be expected to reduce protein levels. Certainly, further investigations including generation of antibody against ApHTT are needed to delineate the full cadre of molecular mechanisms of ApHTT’s role in long-term synaptic plasticity including the aforementioned possibilities. In conclusion, we find that ApHTT is induced following 5 pulses of 5-HT treatment that leads to LTF, a cellular correlate of behavioral sensitization of the *Aplysia* gill-withdrawal reflex and that learning-related regulation of mRNA levels of ApHTT in both presynaptic sensory neurons and postsynaptic motor neurons is important for long-term memory storage.

## Materials and Methods

### Ethics statement

The Institutional Biosafety Committee of The Scripps Research Institute (TSRI) has approved all of the experimental protocols (IBC Protocol 2010-019R1) described in this manuscript. There are no ethical approvals required for the research using invertebrate animals, such as the marine snail *Aplysia*. We have discussed the details of the experiments with the Institutional Animal Care and Use Committee of TSRI and Columbia University Medical Center, and every effort was made to lessen any distress of *Aplysia*.

### mRNA *in situ* hybridization and imaging

A 400 base pair fragment from the start site of the ApHTT ORF was cloned into the EcoRI/XhoI site of the PCR TOPO II vector, linearized with EcoR1 and transcribed with T7 RNA polymerase (Roche, Basel, Switzerland) in the presence of digoxigenin (DIG) RNA labeling mix following the manufacturer’s instructions to make an ApHTT antisense probe. For the sense probe, ApHTT-PCR TOPO II was linearized with Xho I and transcribed with SP6 RNA polymerase. After DNAse I treatment, the sense and antisense probes were used for *in situ* hybridization. A small aliquot (2 µl) was run on 1.5% agarose gel to confirm the integrity of RNA probes. About 1 ng of labeled RNA per µl of hybridization solution was used per culture dish. Sensory-to-motor neuron co-cultures were washed with artificial seawater and fixed for 10 minutes at room temperature with 2 ml of 4% paraformaldehyde in artificial seawater and washed three times in PBS. The *in situ* hybridization was followed as described in Giustetto et al (2003). After hybridization the sense and antisense RNAs were visualized using a Fluorescent Antibody Enhancer kit (Roche, Basel, Switzerland) for DIG detection. Images were acquired using a Zeiss LSM 780 confocal microscope system with 10X/63X objective. Mean fluorescence intensities were measured using NIH IMAGE J and corresponding background signal was subtracted from each mean fluorescence intensities. For the neurite analyses, we randomly selected regions that are minimum of 100 µm away from the initial segment. Percentage change of fluorescence intensity between the control and 5-HT treated neurons were calculated. In all the figures, only projection images are shown.

### Gene expression analysis

Following five pulses of 5-HT treatment (0 minute, 30 minutes and 90 minutes after 5-HT treatment), total RNA was isolated from sensory neuron clusters of *Aplysia* pleural ganglia using the Trizol-chloroform method. The RNA pellet was resuspended in nuclease-free water. RNA concentration and quality was measured using Nanodrop (Thermo Scientific, Waltham, MA). cDNA was generated by reverse transcription from 1 µg of RNA using Quanta cDNA supermix (Quanta Biosciences) according to the manufacturer’s instructions. All qRTPCR primers were synthesized by Integrated DNA technologies. The following primers were used for ApHTT: Ap-Htt-F2 5′-TGGACACTCAGACCACCAGT-3′ and Ap-Htt-R2 5′-CTCTAATAACGCTGCACGGA-3′; for ApC/EBP: ApC/EBP-F1 5′-AGTATCATCCTGTGCCCTCACT-3′ and ApC/EBP-R1 5′-CTGCCTGTGGATGAAACTGTAG-3′; and for 18S rRNA control: Ap18S-F 5′-GTTCACTGCCCGTATCTCCT-3′ and Ap18S-R 5′-AGGCCTGCTTTGAACACTCT-3′. The expressions of ApHTT were first studied by qRTPCR with Power using SYBR green PCR master mix (Applied Biosystems Carlsbad, CA) and then used for the quantification of transcripts. All of the qRTPCR amplifications were performed in a total volume of 10 µl containing 2 µl of H_2_O, 2 µl of cDNA, 5 µl of 2X Master Mix, 1.0 µl each of forward and reverse primers (10 µM) designed based on the ApHTT sequence available at NCBI (http://www.ncbi.nlm.nih.gov/). The qRTPCR reaction was carried out in a 7900 HT Fast Real-Time PCR System (Applied Biosystems) under the following conditions: 95°C for 10 minutes, followed by 40 cycles of 95°C for 15 seconds, 60°C for 1 minutes. Quantification of the target transcripts was normalized to the *Aplysia* 18S rRNA reference gene.

### Microinjection of oligonucleotides to *Aplysia* neurons

Oligonucleotides were synthesized by Integrated DNA Technologies and were gel purified. The following oligonucleotides were used: ApHTT antisense: 5′ g*c*g* tct tca tct cct aaa a*g*a* g 3′, ApHTT sense: 5′ c*t*c* ttt tag gag atg aag a*c*g* c 3′. Both antisense and sense oligonucleotides were phosphothio-modified (indicated by “*” sign) to enhance their stability in the cell. We dissolved oligonucleotides (50 ng/µl) in a buffer containing 0.1% fast green, 10 mM Tris-Cl (pH 7.3), and 250 mM KCl. They were injected under visual guidance into the cytoplasm of *Aplysia* neurons by applying positive air pressure through a picospritzer.

### Electrophysiological assessment of LTF and STF in sensory-to-motor neuron co-cultures

We prepared *Aplysia* sensory-to-motor neuron co-cultures and measured excitatory postsynaptic potentials (EPSPs) as previously described [33]. We evoked the EPSP in L7 motor neuron by stimulating the sensory neuron with a brief depolarizing stimulus using an extracellular electrode. The motor neuron was held at a potential of –30 mV below its resting potential to prevent eliciting action potentials. The synapses with initial EPSPs less than 4 mV were not used for analysis. To induce LTF, we treated cultures with five 5 minutes pulses of 5-HT (10 µM) at 20 minutes intervals. Then, the cultures were maintained at 18°C and the EPSPs were again measured at 24 hours after the initial EPSP measurement. To induce STF, we treated cultures with one 5 minutes pulse of 5-HT (10 µM) after the initial EPSP measurement. EPSP was measured again at 5 minutes after 5-HT treatment.

### Statistical Analysis

Results are denoted as means ± SEM. We used a paired or unpaired Student’s t test to determine statistical significance between two data sets, and one-way ANOVA followed by Tukey HSD post-hoc test to determine statistical significance for multiple comparisons using Graphpad Prism. The statistical significance was indicated by *p<0.05, **p<0.01, or ***p<0.001.
